# Effect of Socioeconomic Status on Mortality after Bacteremia in Working-Age Patients. A Danish Population-Based Cohort Study

**DOI:** 10.1371/journal.pone.0070082

**Published:** 2013-07-25

**Authors:** Kristoffer Koch, Mette Nørgaard, Henrik Carl Schønheyder, Reimar Wernich Thomsen, Mette Søgaard, the Danish Collaborative Bacteremia Network (DACOBAN)

**Affiliations:** 1 Department of Clinical Microbiology, Aalborg University Hospital, Aalborg, Denmark; 2 Department of Clinical Epidemiology, Aarhus University Hospital, Aarhus, Denmark; University of Cambridge, United Kingdom

## Abstract

**Objectives:**

To examine the effect of socioeconomic status (SES) on mortality in patients with bacteremia and the underlying factors that may mediate differences in mortality.

**Methods:**

We conducted a population-based cohort study in two Danish regions. All patients 30 to 65 years of age with first time bacteremia from 2000 through 2008 were identified in a population-based microbiological bacteremia database (n = 8,653). Individual-level data on patients’ SES (educational level and personal income) and comorbid conditions were obtained from public and medical registries. We used Cox regression to examine mortality within 30 days after bacteremia with and without cumulative adjustment for potential mediators.

**Results:**

Bacteremia patients of low SES were more likely to live alone and be unmarried than patients of high SES. They also had more pre-existing comorbidity, more substance abuse, more Staphylococcus aureus and nosocomial infections, and more admissions to small nonteaching hospitals. Overall, 1,374 patients (15.9%) died within 30 days of follow-up. Patients of low SES had consistently higher mortality after bacteremia than those of high SES crude hazard ratio for low vs. high education, 1.38 [95% confidence interval (CI), 1.18–1.61]; crude hazard ratio for low-income vs. high-income tertile, 1.58 [CI, 1.39–1.80]. Adjustment for differences in social support, pre-existing comorbidity, substance abuse, place of acquisition of the infection, and microbial agent substantially attenuated the effect of SES on mortality (adjusted hazard ratio for low vs. high education, 1.15 [95% CI, 0.98–1.36]; adjusted hazard ratio for low-income vs. high-income tertile, 1.29 [CI, 1.12–1.49]). Further adjustment for characteristics of the admitting hospital had minimal effect on observed mortality differences.

**Conclusions:**

Low SES was strongly associated with increased 30-day mortality after bacteremia. Less social support, more pre-existing comorbidity, more substance abuse, and differences in place of acquisition and agent of infection appeared to mediate much of the observed disparities in mortality.

## Introduction

Bacteremia is an increasingly prevalent and life-threatening condition with a reported 30-day mortality above 15% in studies from industrialized countries [1], [2]. In addition to increased risk of infections, low socioeconomic status (SES) may also worsen infection outcomes [3]–[5]. However, few studies have examined the association between SES and mortality after a severe infection, including after bacteremia.

A recent US study of patients hospitalized for sepsis adjusted for demographic factors and pre-existing comorbidity and found that, compared with married patients, widowed, single, and legally separated patients had greater odds of in-hospital death [6]. In another US study, Mendu et al. found an unadjusted relationship between neighborhood poverty rate and mortality within 1 year after bacteremia in patients admitted to intensive care units [7]. In contrast, in a population-based study from New Zealand, Huggan et al. found no relationship between an area-based measure of SES and mortality after *Staphylococcus aureus* bacteremia [8]. Thus, these previous studies have reached conflicting conclusions and used marital status as a proxy for SES or area-based measures of SES, with no data on detailed individual-level measures of SES. Moreover, none of them examined which prognostic factors may mediate socioeconomic disparities in mortality.

Compared with patients of higher SES, patients of lower SES tend to experience less social support, which may lead to more severe infection at admission and a more severe prognosis [6], [9], [10]. Several studies have documented the adverse impact of pre-existing comorbidity and conditions related to substance abuse on survival after bacteremia [11]–[13]. Furthermore, treatment in hospitals with high patient volume and teaching status may be associated with improved outcome and patients of high SES may have a better chance of being admitted to large university hospitals [14], [15].

To examine the effect of SES on mortality after bacteremia, we designed a population-based cohort study. We used two different individual-level indicators of SES, educational level and personal income, to capture different aspects of socioeconomic stratification. We further evaluated if differences in social support, pre-existing comorbidity, substance abuse, infection characteristics, and characteristics of the admitting hospital could explain socioeconomic differences in mortality after bacteremia.

## Methods

### Study Design

We conducted this study as a population-based cohort study. The geographic area included two Danish regions (North Denmark Region and Capital Region) with a total population of 1.7 million persons. All hospitalized patients aged 30 to 65 years with first time bacteremia from 2000 through 2008 were included in the cohort.

### Setting

The Danish tax-funded welfare system provides free access to health care, education, and benefits such as pensions and unemployment coverage. All citizens are granted free services at general practitioners and public hospitals. Only 1% of hospital beds are in the private sector.

Since April 1, 1968, all citizens in Denmark have been registered in the Civil Registration System. A unique personal identification number (civil registration number) allows accurate linkage of information among national registries, including medical registries.

### Identification of Patients with Bacteremia

We obtained data from the Danish Collaborative Bacteremia Network (DACOBAN). This network includes the Departments of Clinical Microbiology in the North Denmark Region (Aalborg Hospital) and the greater Copenhagen area (Hvidovre Hospital and Herlev Hospital). DACOBAN was established to enable coordinated surveillance of all cases of bacteremia in the two regions and to study risk factors and prognostic factors for bacteremia [16]. The three departments that serve these regions record data on all microbiological specimens, including blood cultures, in an electronic laboratory information system (ADBakt, Autonik, Ramsta, Sweden). A research database that consists of all patients with a first-time diagnosis of bacteremia between January 1, 2000 and December 31, 2008 has been established from data from these laboratory information systems. Bacteremia was defined as bacterial or fungal growth in blood cultures where contamination had been ruled out. Coagulase-negative staphylococci, Corynebacterium spp., Bacillus spp. and Propionibacterium acnes were regarded as contaminants unless they were isolated from two or more separate blood culture sets within a 5-day period [17].

We only included patients in the age group of 30 to 65 years because we assumed that most of them had completed their education and were of working age. The civil registration number, age, sex, the date on which the first positive blood culture was drawn (date of bacteremia diagnosis), hospital and specialty, microbial agent, and place of acquisition of infection (community-acquired or nosocomial) were included in the database for all patients. Bacteremia was defined as community-acquired if the first positive blood culture was obtained within 48 hours after hospital admission and as nosocomial if it was drawn more than 48 hours after hospital admission.

### Socioeconomic Status

SES was based on patients’ educational level and personal income. Although the two indicators are related they measure different aspects of socioeconomic stratification. Formal education is normally completed in young adulthood and will therefore to some extent measure early life SES. In contrast, income can change over a life course, but may better capture aspects of SES later in life [18], [19]. To assess the effect of both early life SES and later life SES on mortality after bacteremia, we examined both SES indicators.

SES data were obtained for all patients through registries maintained by the government agency Statistics Denmark [20]–[22]. These registries contain detailed individual-level socioeconomic data, updated yearly, for all Danish citizens. Information on patients’ highest completed education was obtained from the Population’s Education Register, which consists of data generated from surveys and from the administrative records of educational institutions. In 2008 the register contained valid information on education for 97% of the Danish population born from 1945 to 1990 [20]. We categorized educational level into primary/lower secondary education (low), upper secondary education (medium), and tertiary education (high) according to the International Standard Classification of Education (1997) [23].

Patients’ personal annual income was all income subject to income taxation (wages and salaries, and all types of benefits and pensions). Income data was obtained from the Income Statistics Register. Data in the register are primarily supplied by tax authorities and the income data are assumed to equal the real income [21]. Personal annual income was adjusted for inflation according to the year 2000 value of the Danish crown (DKK) and was grouped into tertiles: low-income (1^st^ tertile), middle-income (2^nd^ tertile) and high-income (3^rd^ tertile).

We also obtained data on patients’ employment status, nationality, cohabitation status, and marital status. Employment status was grouped into employed/self-employed, unemployed/employment subsidized by labor market arrangement and early retirement pensioners. We used cohabitation status and marital status as markers for social support. For all variables, we used data from the year preceding the index date of the bacteremia diagnosis.

### Pre-existing Comorbidity

We obtained data from the Danish National Patient Registry for all diagnoses recorded from the start of the registry until the date of bacteremia diagnosis. The registry contains information for almost 100% of all inpatient admissions to public and private non-psychiatric hospitals in Denmark since 1977 and from outpatient and emergency room visits since 1995. Each record includes one primary diagnosis and up to 20 secondary diagnoses, which have been classified according to the International Classification of Diseases [24].

Pre-existing comorbidity was summarized according to the Charlson Comorbidity Index, which was originally developed to predict 1-year mortality in hospitalized medical patients [25]. Since then, the index has been adapted and validated for use with hospital diagnoses and has been used in previous studies of the association between comorbidity and survival after bacteremia [13], [26], [27]. The index consists of 19 disease groups and each disease group is assigned a specific weight depending on the severity of the pre-existing condition. Based on the Charlson index scores three levels of comorbidity were defined: 0 (low), corresponding to patients with no recorded pre-existing comorbidity; 1–2 (medium), and >2 (high).

Diagnoses related to substance abuse (alcohol and drug abuse) are not included in the Charlson index and may influence prognosis after bacteremia. Therefore, we also collected data on previous alcohol- and drug-abuse-related diagnoses from the National Patient Registry.

### Hospital Characteristics

Patients were treated in 1 of 16 public hospitals. We characterized these hospitals according to number of hospital beds, hospital volume, and medical school affiliation. We categorized hospital beds, setup and staffed for use, as less than 300 beds or 300 beds or more. Hospital volume was defined as the annual number of bacteremia patients treated at the institution and categorized as low-volume (≤99 bacteremia patients treated per year), medium-volume (100–299 per year), and high-volume (≥300 per year) hospitals. Teaching hospitals were defined as hospitals directly affiliated with a medical school.

### Follow-up and Mortality

Our outcome was all-cause mortality within 30 days after the bacteremia diagnosis. We obtained data on each patient’s vital status from the Danish Civil Registration System [28]. This registry contains daily updated information on all changes in vital status and migration for all Danish citizens. Patients were followed from the date of their diagnosis to the time of death, date of emigration, or completion of 30 days of follow-up, whichever occurred first.

### Statistical Analysis

We first constructed contingency tables to provide information on baseline characteristics and crude outcomes according to SES, which were inferred on the basis of patients’ educational level and personal income. Using Kaplan-Meier plots we examined mortality within 30 days after bacteremia according to SES.

A Cox proportional hazards model was constructed to determine the association between SES and 30-day mortality. We performed a sequential cumulative adjustment analysis to assess whether differences in social support (cohabitation and marital status), pre-existing comorbidity (comorbidity included in the Charlson Comorbidity Index and conditions related to substance abuse), infection characteristics (place of acquisition, microbial agent, and admitting specialty) or hospital characteristics (number of hospital beds, hospital volume, and medical school affiliation) accounted for socioeconomic differences in mortality. We included the potential mediators in our analyses in a sequence that reflected the temporal relation of the potential mediators (e.g., we assumed that SES would normally precede comorbidity existing at the time of the bacteremia diagnosis).

Log-minus-log plots were used to confirm that the proportional hazards assumption was not violated [29]. All analyses were performed with Stata statistical software, version 11.2 (StataCorp, College Station, TX). The study was approved by the Danish Data Protection Agency (Record no. 2010-41-5650). Informed consent was not required by Danish law.

## Results

### Patient Characteristics

The cohort consisted of 8,653 hospitalized patients aged 30 to 65 years with a first time diagnosis of bacteremia, which corresponded to an incidence of 114 episodes per 100,000 person-years in our study population. The median age of the cohort was 55 years (interquartile range, 47 to 61), and 3,853 (44.5%) were women. Table 1 shows baseline characteristics according to educational level. Only small differences with respect to age and gender were seen. Patients with higher education were slightly younger. The medium-educated patients were more likely to be male. On average, patients with lower education were much less affluent than those with higher education (i.e., 44.7% vs. 17.4% in the lowest income tertile). They were more likely to live alone and be unmarried, and were substantially more likely to be out of the workforce (70.6%) than the patients with higher education (34.6%). Virtually all pre-existing comorbidities were more prevalent among patients with lower education. Only solid cancer, leukaemia, and lymphoma were more prevalent among those with a higher education. Similarly, the prevalence of conditions related to alcohol and drug abuse were substantially increased among those with lower education. Patients with lower education were also more likely to have *Staphylococcus aureus* bacteremia, to have a nosocomial infection, and to receive intensive care. In addition, patients with lower education were more likely to be admitted to small, low-volume, and nonteaching hospitals.

**Table 1:** Baseline characteristics of 8,382 patients with bacteremia, aged 30 to 65 years, categorized according to educational level.

	Educational level					
	Low		Medium		High	
Variable	(n = 3,457; 41.2%)		(n = 3,312; 39.5%)		(n = 1,613; 19.2%)	
**Demographic charateristic**						
Median age, *y*	56		56		54	
Men, n (%)	1,770	(51.2)	2,041	(61.6)	841	(52.1)
*Nationality, n (%)*						
Danish	3,187	(92.2)	3,057	(92.3)	1,448	(89.8)
Immigrants from Western countries	72	(2.1)	102	(3.1)	67	(4.2)
Immigrants from non-Western countries	198	(5.7)	153	(4.6)	98	(6.1)
**Socioeconomic indicators, n (%)**						
*Income category*						
Low (1^st^ tertile)	1,544	(44.7)	965	(29.1)	281	(17.4)
Middle (2^nd^ tertile)	1,309	(37.9)	1,145	(34.6)	335	(20.8)
High (3^rd^ tertile)	600	(17.4)	1,194	(36.1)	995	(61.7)
Data missing	4	(0.1)	8	(0.2)	2	(0.1)
*Employment*						
Employed/self-employed	1,007	(29.1)	1,693	(51.1)	1,049	(65.0)
Unemployed/labor market arrangement	910	(26.3)	793	(23.9)	309	(19.2)
Early retirement pension	1,530	(44.3)	812	(24.5)	249	(15.4)
Data missing	10	(0.3)	14	(0.4)	6	(0.4)
**Social support, n (%)**						
*Living alone*						
Yes	1,788	(51.7)	1,325	(40.0)	581	(36.0)
No	1,669	(48.3)	1,987	(60.0)	1,032	(64.0)
*Marital status*						
Married	1,458	(42.2)	1,826	(55.1)	971	(60.2)
Divorced or widowed	1,006	(29.1)	833	(25.2)	317	(19.7)
Never married	983	(28.4)	639	(19.3)	319	(19.8)
Data missing	10	(0.3)	14	(0.4)	6	(0.4)
**Pre-existing comorbidity, n (%)**						
*Clinical conditions included in the CCI*						
Previous myocardial infarction	207	(6.0)	185	(5.6)	64	(4.0)
Congestive cardiac insufficiency	213	(6.2)	157	(4.7)	53	(3.3)
Peripheral vascular disease	236	(6.8)	192	(5.8)	62	(3.8)
Cerebrovascular disease	332	(9.6)	330	(10.0)	113	(7.0)
Dementia	36	(1.0)	44	(1.3)	13	(0.8)
Hemiplegia	49	(1.4)	28	(0.9)	11	(0.7)
Chronic pulmonary disease	475	(13.7)	297	(9.0)	98	(6.1)
Connective tissue disease	146	(4.2)	142	(4.3)	58	(3.6)
Peptic ulcer disease	450	(13.0)	333	(10.1)	81	(5.0)
Mild liver disease	454	(13.1)	364	(11.0)	99	(6.1)
Moderate or severe liver disease	212	(6.1)	166	(5.0)	53	(3.3)
Diabetes, without complications	488	(14.1)	398	(12.0)	140	(8.7)
Diabetes with complications	311	(9.0)	248	(7.5)	86	(5.3)
Moderate or severe kidney disease	264	(7.6)	282	(8.5)	91	(5.6)
Solid cancer	590	(17.1)	628	(19.0)	312	(19.3)
Metastatic solid cancer	135	(3.9)	171	(5.2)	97	(6.0)
Leukemia	47	(1.4)	74	(2.2)	37	(2.3)
Lymphoma	102	(3.0)	160	(4.8)	98	(6.1)
HIV/AIDS	67	(1.9)	24	(0.7)	6	(0.4)
*Charlson comorbidity index score*						
Low (0)	1,103	(31.9)	1,182	(35.7)	733	(45.4)
Medium (1–2)	1,291	(37.3)	1,182	(35.7)	507	(31.4)
High (>2)	1,063	(30.8)	948	(28.6)	373	(23.1)
**Substance abuse, n (%)**						
Alcohol abuse	719	(20.8)	624	(18.8)	192	(11.9)
Drug abuse	344	(10.0)	115	(3.5)	38	(2.4)
**Characteristics of infection, n (%)**						
*Microbial agent*						
* Staphylococcus aureus*	565	(16.3)	546	(16.5)	226	(14.0)
* Streptococcus pneumoniae*	394	(11.4)	375	(11.3)	190	(11.8)
Other gram-positive organisms	645	(18.7)	641	(19.4)	356	(22.1)
* Escherichia coli*	866	(25.1)	803	(24.3)	411	(25.5)
Other enterobacteria	1,302	(37.7)	1,206	(36.4)	609	(37.8)
Other gram-negative organisms	268	(7.8)	295	(8.9)	126	(7.8)
Polymicrobial or fungal	396	(11.5)	384	(11.6)	172	(10.7)
*Acquisition*						
Community-acquired	2,175	(62.9)	2,089	(63.1)	1,054	(65.3)
Nosocomial	1,266	(36.6)	1,207	(36.4)	550	(34.1)
Data missing	16	(0.5)	16	(0.5)	9	(0.6)
*Specialty*						
Internal medicine	2,269	(65.6)	2,110	(63.7)	996	(61.8)
Surgery	847	(24.5)	899	(27.1)	495	(30.7)
Intensive care	327	(9.5)	286	(8.6)	108	(6.7)
Data missing	14	(0.4)	17	(0.5)	14	(0.9)
**Hospital characteristics, n (%)**						
*Bed size*						
Low (<300 beds)	725	(21.0)	601	(18.2)	242	(15.0)
High (>300 beds)	2,732	(79.0)	2,711	(81.9)	1,371	(85.0)
*Hospital volume* a						
Low (≤99/year)	678	(19.6)	561	(16.9)	271	(16.8)
Medium (100–299/year)	517	(15.0)	492	(14.9)	190	(11.8)
High (≥300/year)	2,262	(65.4)	2,259	(68.2)	1,152	(71.4)
*Teaching hospital* b						
No	497	(14.4)	404	(12.2)	177	(11.0)
Yes	2,960	(85.6)	2,908	(87.8)	1,436	(89.0)
**Mortality, n (%)**						
30-day	602	(17.4)	529	(16.0)	209	(13.0)

Abbreviation: CCI, Charlson comorbidity index.

a Hospital volume was defined as the annual number of bacteremia patients treated at the institution.

b Teaching hospitals were defined as hospitals directly affiliated with a medical school.

A similar but more extreme pattern of differences was seen for income categories: bacteremia patients with low versus high income were 1.5 times more likely to live alone and be unmarried, had a 1.5 to 4 times higher prevalence of many comorbidities, and had a more than 4-fold higher risk of substance abuse (Table 2).

**Table 2:** Baseline characteristics of 8,633 patients with bacteremia, aged 30 to 65 years, categorized according to income.

	Income category					
	Low		Middle		High	
Variable	(1^st^ tertile; n = 2,878)		(2^nd^ tertile; n = 2,878)		(3^rd^ tertile; n = 2,877)	
**Demographic charateristic**						
Median age, *y*	55		55		55	
Men, n (%)	1,450	(50.4)	1,481	(51.5)	1,858	(64.6)
*Nationality, n (%)*						
Danish	2,524	(87.7)	2,633	(91.5)	2,713	(94.3)
Immigrants from Western countries	109	(3.8)	78	(2.7)	84	(2.9)
Immigrants from non-Western countries	245	(8.5)	167	(5.8)	80	(2.8)
**Socioeconomic indicators, n (%)**						
*Educational level*						
Low	1,515	(52.6)	1,320	(45.9)	618	(21.5)
Medium	943	(32.8)	1,138	(39.5)	1,223	(42.5)
High	274	(9.5)	337	(11.7)	1,000	(34.8)
Data missing	146	(5.1)	83	(2.9)	36	(1.3)
*Employment*						
Employed/self-employed	412	(14.3)	1,023	(35.6)	2,367	(82.3)
Unemployed/labor market arrangement	843	(29.3)	995	(34.6)	260	(9.0)
Early retirement pension	1,605	(55.8)	858	(29.8)	248	(8.6)
Data missing	18	(0.6)	2	(0.1)	2	(0.1)
**Social support, n (%)**						
*Living alone*						
Yes	1,513	(52.6)	1,428	(49.6)	923	(32.1)
No	1,365	(47.4)	1,450	(50.4)	1,954	(67.9)
*Marital status*						
Married	1,239	(43.1)	1,315	(45.7)	1,789	(62.2)
Divorced or widowed	883	(30.7)	775	(26.9)	578	(20.1)
Never married	738	(25.6)	786	(27.3)	508	(17.7)
Data missing	18	(0.6)	2	(0.1)	2	(0.1)
**Pre-existing comorbidity, n (%)**						
*Clinical conditions included in the CCI*						
Previous myocardial infarction	170	(5.9)	161	(5.6)	135	(4.7)
Congestive cardiac insufficiency	167	(5.8)	157	(5.5)	115	(4.0)
Peripheral vascular disease	207	(7.2)	178	(6.2)	117	(4.1)
Cerebrovascular disease	310	(10.8)	284	(9.9)	207	(7.2)
Dementia	40	(1.4)	39	(1.4)	14	(0.5)
Hemiplegia	21	(0.7)	48	(1.7)	24	(0.8)
Chronic pulmonary disease	397	(13.8)	328	(11.4)	175	(6.1)
Connective tissue disease	138	(4.8)	128	(4.5)	93	(3.2)
Peptic ulcer disease	406	(14.1)	323	(11.2)	167	(5.8)
Mild liver disease	498	(17.3)	321	(11.2)	135	(4.7)
Moderate or severe liver disease	227	(7.9)	160	(5.6)	61	(2.1)
Diabetes, without complications	436	(15.2)	378	(13.1)	238	(8.3)
Diabetes with complications	285	(9.9)	238	(8.3)	136	(4.7)
Moderate or severe kidney disease	235	(8.2)	238	(8.3)	180	(6.3)
Solid cancer	430	(14.9)	500	(17.4)	624	(21.7)
Metastatic solid cancer	100	(3.5)	138	(4.8)	169	(5.9)
Leukemia	40	(1.4)	55	(1.9)	66	(2.3)
Lymphoma	70	(2.4)	114	(4.0)	177	(6.2)
HIV/AIDS	62	(2.2)	32	(1.1)	13	(0.5)
*Charlson comorbidity index score*						
Low (0)	872	(30.3)	981	(34.1)	1,259	(43.8)
Medium (1–2)	1,064	(37.0)	1,035	(36.0)	976	(33.9)
High (>2)	942	(32.7)	862	(30.0)	642	(22.3)
**Substance abuse, n (%)**						
Alcohol abuse	829	(28.8)	526	(18.3)	235	(8.2)
Drug abuse	338	(11.7)	148	(5.1)	45	(1.6)
**Characteristics of infection, n (%)**						
*Microbial agent*						
* Staphylococcus aureus*	513	(17.8)	463	(16.1)	409	(14.2)
* Streptococcus pneumoniae*	308	(10.7)	295	(10.3)	384	(13.4)
Other gram-positive organisms	556	(19.3)	547	(19.0)	582	(20.2)
* Escherichia coli*	694	(24.1)	749	(26.0)	693	(24.1)
Other enterobacteria	1,031	(35.8)	1,108	(38.5)	1,066	(37.1)
Other gram-negative organisms	223	(7.8)	253	(8.8)	230	(8.0)
Polymicrobial or fungal	345	(12.0)	318	(11.1)	322	(11.2)
*Acquisition*						
Community-acquired	1,766	(61.4)	1,831	(63.6)	1,880	(65.4)
Nosocomial	1,096	(38.1)	1,036	(36.0)	983	(34.2)
Data missing	16	(0.6)	11	(0.4)	14	(0.5)
*Specialty*						
Internal medicine	1,857	(64.5)	1,862	(64.7)	1,830	(63.6)
Surgery	719	(25.0)	745	(25.9)	835	(29.0)
Intensive care	289	(10.0)	254	(8.8)	196	(6.8)
Data missing	13	(0.5)	17	(0.6)	16	(0.6)
**Hospital characteristics, n (%)**						
*Bed size*						
Low (<300 beds)	575	(20.0)	607	(21.1)	443	(15.4)
High (>300 beds)	2,303	(80.0)	2,271	(78.9)	2,434	(84.6)
*Hospital volume* a						
Low (≤99/year)	552	(19.2)	559	(19.4)	457	(15.9)
Medium (100–299/year)	417	(14.5)	434	(15.1)	383	(13.3)
High (≥300/year)	1,909	(66.3)	1,885	(65.5)	2,037	(70.8)
*Teaching hospital* b						
No	377	(13.1)	437	(15.2)	291	(10.1)
Yes	2,501	(86.9)	2,441	(84.8)	2,586	(89.9)
**Mortality, n (%)**						
30-day	566	(19.7)	433	(15.1)	371	(12.9)

Abbreviation: CCI, Charlson comorbidity index.

a Hospital volume was defined as the annual number of bacteremia patients treated at the institution.

b Teaching hospitals were defined as hospitals directly affiliated with a medical school.

### Mortality

**Figure 1:**
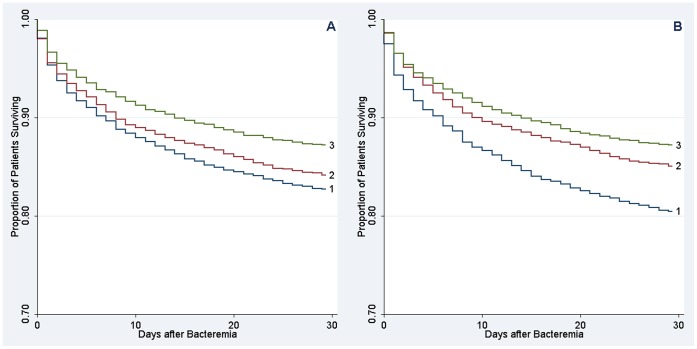
Crude Kaplan-Meier survival curves according to socioeconomic status. A) Educational level (low 1, medium 2, high 3), B) Income (low 1, middle 2, high 3).

Overall, 1,374 patients (15.9%) died within 30 days of follow-up. There was a substantial gradient in mortality according to both educational level and income categories. Survival curves for the different levels of education and income diverged early after the bacteremia diagnosis and the differences in mortality persisted throughout the 30 days of follow-up (Figure 1). The 30-day mortality among the lower educated patients was 17.4% compared with 13.0% for those with higher education, which was an absolute difference in 30-day mortality of 4.5% [95% confidence interval (CI): 2.4–6.5%] and corresponded to a crude hazard ratio of 1.38 [95% CI: 1.18–1.61]. The difference in 30-day mortality was 6.7% [95% CI: 4.8–8.6%] when patients in the low-income tertile (30-day mortality, 19.7%) were compared with patients in the high-income tertile (30-day mortality, 12.9%). This corresponded to a crude hazard ratio of 1.58 [95% CI: 1.39–1.80].

Sequential adjustment for social support, pre-existing comorbidity, substance abuse, and infection characteristics attenuated the effect of SES on mortality (Table 3 and 4). Further adjustment for differences in characteristics of the admitting hospital had only a marginal impact on the adjusted 30-day mortality hazard ratios. The fully adjusted risk estimates showed that there was still a residual difference in mortality according to both educational level (low vs. high education, 1.14 [95% CI: 0.97–1.35] and income categories (low vs. high income, 1.30 [95% CI: 1.13–1.49] after adjustment for a range of known prognostic factors.

**Table 3:** 30-day mortality risk after first time diagnosis of bacteremia according to educational level and effect of adjustment for social support, pre-existing comorbidity, substance abuse, characteristics of infection, and hospital characteristics.

	Educational level		
	Low	Medium	High
Unadjusted	1.38 (1.18–1.61)	1.25 (1.07–1.47)	1.00 (reference)
Adjusted			
Demographiccharacteristics^a^	1.33 (1.14–1.56)	1.17 (0.99–1.37)	1.00 (reference)
+ social support^b^	1.27 (1.08–1.49)	1.15 (0.98–1.35)	1.00 (reference)
+ pre-existingcomorbidity^c^	1.20 (1.02–1.41)	1.08 (0.92–1.28)	1.00 (reference)
+ characteristics ofinfection^d^	1.15 (0.98–1.36)	1.04 (0.88–1.22)	1.00 (reference)
+ hospitalcharacteristics^e^	1.14 (0.97–1.35)	1.03 (0.88–1.22)	1.00 (reference)

a Age, sex, and nationality.

b Cohabitation and marital status.

c Comorbidities included in the Charlson comorbidity index and conditions related to substance abuse.

d Microbial agent, place of acquisition, and admitting specialty.

e Number of hospital beds, hospital volume, and medical school affiliation.

**Table 4:** 30-day mortality risk after first time diagnosis of bacteremia according to income and effect of adjustment for social support, pre-existing comorbidity, substance abuse, characteristics of infection, and hospital characteristics.

	Income Category		
	Low (1^st^ tertile)	Middle (2^nd^ tertile)	High (3^rd^ tertile)
Unadjusted	1.58 (1.39–1.80)	1.18 (1.02–1.35)	1.00 (reference)
Adjusted			
Demographic characteristics^a^	1.69 (1.48–1.93)	1.22 (1.07–1.41)	1.00 (reference)
+ social support^b^	1.58 (1.38–1.81)	1.16 (1.01–1.33)	1.00 (reference)
+ pre-existing comorbidity^c^	1.37 (1.19–1.57)	1.08 (0.92–1.22)	1.00 (reference)
+ characteristics of infection^d^	1.29 (1.12–1.49)	1.03 (0.89–1.18)	1.00 (reference)
+ hospital characteristics^e^	1.30 (1.13–1.49)	1.03 (0.89–1.19)	1.00 (reference)

a Age, sex, and nationality.

b Cohabitation and marital status.

c Comorbidities included in the Charlson comorbidity index and conditions related to substance abuse.

d Microbial agent, place of acquisition, and admitting specialty.

e Number of hospital beds, hospital volume, and medical school affiliation.

To examine whether income mediated the effect of education, we also included income as a covariate in our statistical model examining the effect of education on mortality. The effect of education on mortality after bacteremia was further attenuated after inclusion of income as a covariate (low vs. high education, 1.08 [95% CI: 0.91–1.28]), showing that income in part mediated the effect of education.

## Discussion

In this population-based cohort study we found that patients of lower SES, inferred on the basis of educational level and income, had higher 30-day mortality after bacteremia than those of higher SES. The association between SES and mortality was most pronounced when we used income as SES indicator. More than half of the socioeconomic differences in mortality could be explained by differences in social support, pre-existing comorbidity, alcohol and drug abuse, and characteristics of the infection. In contrast, characteristics of the admitting hospital had only a marginal explanatory effect on the socioeconomic mortality differences. Our findings thus suggest that socioeconomic disparities in mortality after bacteremia are to a large extent explained by a range of adverse prognostic factors that are present before hospital admission and include severe pre-existing comorbidity, unhealthy lifestyle, and lack of social support.

For the clinician it is important to know if certain groups of patients with severe infection have a poorer prognosis than others and our study therefore has clinical implications. Worse prognosis among bacteremia patients of lower SES imply that these patients would benefit from increased clinical attention. Our findings of a much higher prevalence of comorbidities among bacteremia patients of lower SES suggest that special attention should be paid to improved management of these patients’ comorbidities, which may reduce their excess mortality risk.

We are aware of only three studies that have examined socioeconomic disparities in mortality in cohorts of patients with sepsis or bacteremia. Seymour et al. conducted a population-based cohort study of 37,524 hospitalizations for sepsis in New Jersey, US [6]. After adjusting for demographic factors and comorbid conditions, they found that single and divorced men and single women had greater odds of in-hospital mortality than married persons. In contrast to our study, this study used marital status as a proxy for social factors and did not include data on more precise measures of SES, such as educational level and income. Furthermore, identification of patients with infections was based on administrative ICD-9-CM codes and the infections were not microbiologically verified. Mendu et al. analyzed data from 2,435 patients with bacteremia who were admitted to intensive care units at two hospitals in Boston, Massachusetts, US [7]. This study reported an unadjusted ‘dose-response’ relationship between neighborhood poverty rate and mortality within one year after bacteremia among patients receiving critical care. Adjustment for demographic factors, patient type, comorbidity, laboratory data, and severity of illness attenuated this association substantially and the authors concluded that neighborhood poverty was not associated with mortality after bacteremia. However, their use of an aggregate area-based measure of SES may have led to some inaccuracy due to misclassification of individual SES. A third study by Huggan et al. included 779 patients with *Staphylococcus aureus* bacteremia admitted to hospitals in Canterbury, New Zealand [8]. The authors reported that there was no relationship between an address-based measure of deprivation and mortality but did not present any estimates. This study also used an area-based measure of SES, which may have resulted in misclassification of individual SES [30].

In contrast with previous studies we used a sequential cumulative adjustment analysis to evaluate a range of recognized prognostic factors as potential mediators of the socioeconomic mortality differences. Even after full adjustment for all the potential mediators included in our study, we still found a residual difference in mortality. It is likely that the residual difference may be explained partly by greater levels of undiagnosed comorbidity among patients of lower SES, which again may be due to poor self-care and late presentation of clinical disease. However, we speculate that unmeasured variation in severity of infection at admission may also explain some of the residual mortality differences. Furthermore, since we did not have precise data on bacteremia patients’ care and treatment, we cannot rule out that difference in the quality of care contributed to the residual mortality differences.

Our study has several important limitations. First, we used information collected during routine clinical work and data from administrative registries, which limited clinical detail in our study. More information on clinical parameters would have enabled us to better characterize severity of the infection and to assess any differences in severity according to SES. On the other hand, the use of routinely collected microbiological data and accurate linkage of high-quality registries enabled us to avoid some major methodological problems, such as selection and surveillance bias. Use of prospectively recorded individual data on SES indicators, which were collected independently, also reduced misclassification of patients SES. Second, our outcome measure was all-cause mortality, and we did not have any data on other important outcomes, such as recovery and functional status. When interpreting all-cause mortality, it must be considered that patients may have died from causes unrelated to their infection. Nevertheless, by including only deaths that occurred within 30 days after the date of bacteremia diagnosis we assume that most deaths would be at least to some extent related to the infection. Third, even though we studied a large cohort of bacteremia patients statistical precision was still limited. Fourth, we included bacteremia patients with a microbiologically confirmed infection, which allowed us to assess the mediating effect of the microbial agent on the association between SES and mortality. We can assume that the vast majority of the bacteremia patients in our cohort fulfilled the criteria for sepsis [31], [32]. However, since less than 50% of patients with sepsis have documented bacteremia, our findings cannot necessarily be generalised to sepsis patients [33], [34].

In conclusion, we found that patients of low SES had higher mortality within 30 days after bacteremia than those of high SES. Differences in social support, pre-existing comorbidity, substance abuse, and characteristics of the infection were important explanatory factors for these SES-mortality gradients. In contrast, characteristics of the admitting hospital seemed to have a negligible role in explaining disparities in mortality. The residual impact of SES on mortality might be explained by differences in bacteremia severity and treatment of the infection. Future studies should assess the importance of potential differences in severity of illness and quality of care in explaining socioeconomic mortality differences after bacteremia.
